# Ten simple rules for developing bioinformatics capacity at an academic institution

**DOI:** 10.1371/journal.pcbi.1009592

**Published:** 2021-12-09

**Authors:** Shaun Aron, C. Victor Jongeneel, Paballo Abel Chauke, Melek Chaouch, Judit Kumuthini, Lyndon Zass, Fouzia Radouani, Samar Kamal Kassim, Faisal M. Fadlelmola, Nicola Mulder

**Affiliations:** 1 Sydney Brenner Institute for Molecular Bioscience, University of the Witwatersrand, Johannesburg, South Africa; 2 Independent consultant, Rolle, Switzerland; 3 Computational Biology Division, Department of Integrative Biomedical Sciences, Institute of Infectious Disease and Molecular Medicine, CIDRI Africa Wellcome Trust Centre, Faculty of Health Sciences, University of Cape Town, Cape Town, South Africa; 4 Laboratory of bioinformatics, biomathematics and biostatistics, Institut Pasteur de Tunis, Université Tunis El Manar, Belvédère, Tunisia; 5 South African National Bioinformatics Institute, University of the Western Cape, Cape Town, South Africa; 6 Chlamydiae & Mycoplasmas Laboratory Research Department, Institut Pasteur du Maroc, Casablanca, Morocco; 7 Medical Biochemistry & Molecular Biology Department, and MASRI Research Institute, Faculty of Medicine, Ain Shams University, Cairo, Egypt; 8 Centre for Bioinformatics and Systems Biology, Faculty of Science, University of Khartoum, Khartoum, Sudan

## Introduction

Bioinformatics is an applied interdisciplinary field whose primary purpose is to develop and deploy computational techniques to store, organize, and aid in the analysis and interpretation of large-scale data obtained from biological systems. While rooted in the analysis of nucleotide and protein sequences, it now encompasses techniques targeting multiple data acquisition modalities and seeks to comprehend the functioning of biological systems at many different levels. Bioinformaticians need to be cognizant of diverse scientific fields: basic and molecular biology, genetics, mathematics, statistics, and computer science at a minimum, thus requiring a thoroughly interdisciplinary set of skills to successfully carry out their duties. Due to the growing importance of bioinformatics in enabling modern biomedical research, programs and core facilities have been established in most academic institutions in the developed world over the last 30 years.

At present, there are relatively few research and higher education institutions in low- and middle-income countries (LMICs) that have incorporated bioinformatics into their academic curricula or are hosting bioinformatics research or service groups [1,2]. There are many reasons for this, including the relative lack of research projects requiring computational analysis, lack of local expertise, lack of required infrastructure, lack of appropriate internal financial support, and the resistance of many academic institutions to the incorporation of new fields of teaching and research [3]. However, there is an increasing recognition in some African countries, and other LMICs, that bioinformatics needs to be developed as an independent discipline, both in the academic curricula and in research portfolios [4,5].

H3ABioNet is a Pan-African network of bioinformaticians, funded by the United States National Institutes of Health (NIH), that aims to provide technical and scientific support to the Human Heredity and Health in Africa (H3Africa) genomics research program [6]. As part of its mission, H3ABioNet has developed training and capacity building projects ranging from introductory courses to advanced workshops on bioinformatics and related topics, and from small infrastructures to major distributed networks [7,8]. H3ABioNet is, therefore, in a good position to help institutions ranging from individual laboratories to institutes, faculties, and universities in developing bioinformatics capacity across the African continent. Below, we propose 10 simple rules for developing bioinformatics at various levels at an institution, based on our experience in working with LMICs. Rule 4 covers several different scenarios depending on the type and level of bioinformatics expertise aimed for.

## Rule 1: Define the exact needs of your institution

It is essential, when thinking about developing a bioinformatics program, to start by asking the question of what is really needed in the local institutional context, who needs it, at what level, and why. Do researchers simply need to acquire some skills to analyze their own data or should bioinformatics skills be established on a larger scale? The fundamental issue is whether a bioinformatics research group or a cadre of well-trained bioinformaticians will address a real perceived need. There could be many potential drivers for such a need, for example, existing research projects generating data for which analysis expertise is lacking, or the creation of an academic unit specializing in genomics and data analysis, algorithms, tools and pipelines development, or a local shortage in the job market, or a long-term funding opportunity for which bioinformatics capacity is required. It is also possible that an opportunity arises to recruit a world-class faculty member with expertise in the field or that the institution has a long-term commitment and support from its existing faculty to build a competitive bioinformatics program. Getting buy-in from the faculty and/or university is one of the main bottlenecks in providing bioinformatics teaching or training and research programs in LMICs. This is a challenge for 3 reasons: Firstly, there must be evidence that there will be an uptake of graduates in the job market once they have completed their studies—this is often a problem as there are not enough employers looking for bioinformaticians in LMICs. Training bioinformaticians whose only job prospects would be to emigrate or to train more bioinformaticians with equally poor prospects is not an attractive proposition. However, this is changing as more LMICs are conducting or participating in large-scale genome projects and providing genomic services. Secondly, many institutions have difficulty in finding appropriately skilled lecturers and trainers due to the lack of local expertise, and thirdly, it takes significant resources to establish a new university accredited course. In some cases, accredited online courses have proven to be of great help in teaching bioinformatics (https://www.coursera.org/courses?query=bioinformatics).

Therefore, we strongly recommend that before deciding to build a bioinformatics training and/or research program, the question be asked of whether this meets clearly expressed institutional needs, provides sustainable career choices, and has the support of the local community, including faculty, students, and researchers. If the answer is positive, the scope of the needs should be narrowed down further:

- Will this be a purely educational program, or will it also include a research component?
- Who will be the target population for the educational program? Undergraduates, postgraduates, or established researchers?
- Will it include a new educational program, or will the courses be integrated into an existing degree or professional development framework?
- For professional development, is there a project or data available on which the training can be based?
- Will it be free of charge or a paid for program and who will fund it?
- Is your primary goal to train professional bioinformaticians or computational scientists, or to provide additional skills to established practitioners in other fields (biology, computer science, biotechnology, etc.)?

## Rule 2: Identify possible funding resources (public and private)

Establishing a bioinformatics training or research program at any level will require some form of funding in order to be actualized. Obtaining funding is critical to scientists for research as well as development endeavors [9]. It is vital that funds get secured immediately after identifying the institutional needs (Rule 1) either through grant applications or other solicitations from both private and public donors. It is important to approach possible funders with a clear proposal and strategy of what is to be achieved in terms of bioinformatics capacity building in the institution as well as the intended outputs and outcomes. One avenue to identify possible funders is to create a tracking system for potential grants to apply for, be it government or private sector based. A third-party funding tracker can be created manually in Excel or online using different platforms to create a database that captures and includes funders in the scientific field, particularly bioinformatics. The tracker can be used to explore each potential funder’s website and sign up for the newsletters, digests, and Twitter or other social media accounts where funding announcements are made. It is useful to sign up to diverse funding hubs such as Research Professional (http://info.researchprofessional.com/research-africa/) and FundsforNGOs (https://www.fundsforngos.org/guides/document-identify-locate-approach-funders/), which allow users to find potential funders and track specific sponsors for certain scientific research endeavor’s such as capacity building and development in science. On such platforms, one can create keywords and search terms that allow the potential applicant to track calls for applications in terms of open dates, eligibility requirements, and deadlines among other important announcements necessary to assist one to apply for and hopefully secure funds.

Two avenues to source funding include, firstly, responding to grant calls that are specifically aimed at developing bioinformatics research or training programs from large international funders, always ensuring that you meet the requirements for the call before applying. Secondly, a “cold” email briefly describing your project plans and outcomes could be sent to the relevant contact person at private and public institutions who may be interested in funding your project. In whatever route you choose to identify and apply for funding sources for your venture, it is important to follow instructions, supply enough information, and pay attention to technical aspects such as spelling, grammar, and punctuation on text or tone when it is on the phone or face-to-face communication. Creating pamphlets, animations, and simplifying complex scientific jargon can go a long way in assisting with the successful solicitation of funding by providing easy-to-explain, digestible nuggets of information for potential funders especially those not familiar with the field of bioinformatics.

## Rule 3: Identify established bioinformatics organizations available to support the determined needs

It is important to plan the path to achieving the selected outcome within a realistic timeline. The plan will depend on what human resources and skills are already available on the ground. For each of the expected outcomes outlined above, there are resources available from recognized international bioinformatics organizations, for example, H3ABioNet, EMBnet, GOBLET, and others that can assist or facilitate the process of achieving that outcome. There are also specific steps an organization can take to move the process forward, as will be highlighted in the following rules. If there is no local expertise at the start of such an endeavor, identifying and gaining the support of a mentor nearby or abroad is crucial. For large-scale analyses, curriculum development, teaching, and supervision, it may be necessary to engage institutional colleagues in the relevant departments such as computer science, mathematics, or statistics. When considering human capacity development, it may be worth reviewing the ISCB competency framework for bioinformatics, which addresses competencies required by different personas [10]. Referencing the ISCB competency framework can also facilitate further refining of the institutions’ needs and desired outcomes and later in the design of a curriculum. The more specific these needs and outcomes, the more detailed the action plan will be.

## Rule 4: Determine the desired outcome and generate a game plan (academic or service orientated)

**Fig 1:**
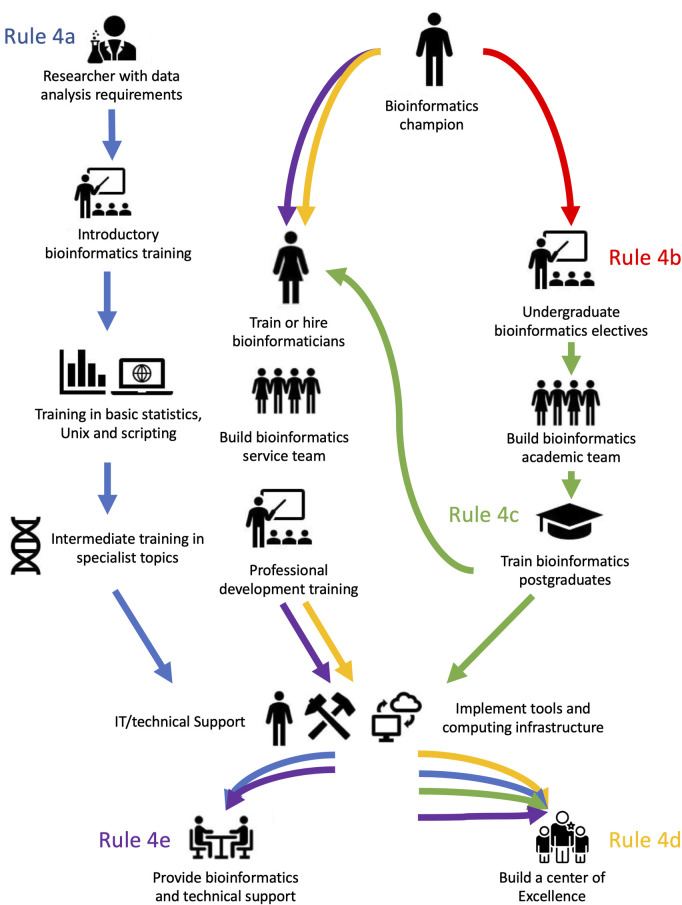
A diagrammatic representation of the scenarios outlined in Rule 4. While each scenario is unique and described in Rules 4a–4e, there is a significant overlap in the paths taken to achieve each outcome.

The next step is to define the desired outcomes of the program and the timeline to achieve those outcomes. The outcomes will of course be driven by the choices made during the determination of the needs, but clarifying them at an early stage will be helpful in informing the scope and timeline of the implementation stage and the resources that will be required to achieve it. Below are some examples of possible outcomes and approximate timelines. Fig 1 demonstrates those outcomes and how they are related:

- Training existing researchers to analyze data (1 year)—see **Rule 4a.**
- Offering bioinformatics electives to undergraduate students (1 year)—see **Rule 4b.**
- Setting up a formal degree program in bioinformatics at the MSc or PhD level where graduates can become trainers/educators and/or researchers themselves (4 years +)—see **Rule 4c.**
- Building a national center of excellence involving multiple faculty hires and the creation of a new academic unit (center, department, institute) (5 to 10 years)—see **Rule 4d**.
- Build up a bioinformatics service to serve a connected bioinformatics community (2 years)—see **Rule 4e.**

The level of resources required to achieve each of these outcomes varies, as will be made clear from the following scenarios. As an illustration, in scenarios 4a to 4e, we provide a brief outline of the possible outcomes and the relevant steps that can be followed to achieve each of these (Fig 1).

### Rule 4a: Train existing students/researchers to analyze data

In this scenario, the targeted students/researchers would have an established background in molecular biology, computer science, statistics, or mathematics, mostly at the postgraduate or junior researcher level. Senior researchers who have started to delve into the application of bioinformatics in their areas of interest could also serve as ideal candidates to drive this specific scenario. An assessment should be conducted to determine the competencies that would need to be developed for the trainees to efficiently analyze datasets produced by related research projects—these competencies will differ based on their background and the types of data needing analysis; therefore, their learning paths may also differ. In most instances, a suggested starting point will be for trainees to attend an introduction to bioinformatics course, such as the one offered by H3ABioNet (https://h3abionet.org/training/ibt) [11]. Introductory courses would provide trainees with basic bioinformatics foundational knowledge as well as foster and encourage further exploration and self-study in areas of interest. Such online approaches do not require local experts, only teaching assistants or advisors to host the classroom, with reasonable knowledge on the subject and the ability to guide trainees through the recommended courses, as the expert lecturers are available online with these course models. This initial course can be followed up by intermediate or advanced courses to develop skills specific to the data analysis needs in the targeted research group [12].

In addition to H3ABioNet (https://www.h3abionet.org/training/courses-and-events; https://egenomics.h3abionet.org/), a number of other organizations also provide online and face-to-face training in a variety of bioinformatics analysis topics, including ELIXIR (https://elixir-europe.org/platforms/training) and GOBLET (https://www.mygoblet.org/training-portal). The Carpentries also provides a number of courses and lecture material in R and Python programming, which would be good follow-up courses, providing participants with the skills necessary for handling and manipulating large datasets and visualization of complex data (https://software-carpentry.org/lessons/; https://datacarpentry.org/lessons/).

Once a core set of individuals have been trained in a specific area of expertise and successfully applied the training in practice, these individuals can be further trained to become trainers and teaching assistants for locally run courses and workshops (https://www.ebi.ac.uk/training/train-trainer; https://carpentries.github.io/instructor-training/; https://www.futurelearn.com/courses/train-the-trainer-design-genomics-training). In this scenario, a vital component will be to negotiate access to, or set up, the appropriate computing infrastructure and data analysis workflows to be able to efficiently analyze large datasets (Rule 6). Coupled with this, there may be a need to enlist the help of trained system administrators or technicians to set up these resources. H3ABioNet has created a list of computational resources available in Africa, guidelines for system administrators, and some data analysis workflows available through the network (https://www.h3abionet.org/computing-infrastructure; https://www.h3abionet.org/tools-and-services/technical-guidelines; https://www.h3abionet.org/tools-and-services/workflows).There are also several resources that can be used to access public datasets if required for training purposes, e.g,. 1000 Genomes Project (https://www.internationalgenome.org/data), EBI BioStudies (previously ArrayExpress) (https://www.ebi.ac.uk/biostudies/), and the European Genome-Phenome Archive (EGA) (https://ega-archive.org/). Over time, these trained individuals may create a local critical mass of bioinformatic scientists capable of providing local or regional support for various types of data analysis.

### Rule 4b: Offer bioinformatics undergraduate electives

This outcome will require a trainer or trainers with adequate bioinformatics knowledge and skills or a bioinformatics champion with the relevant bioinformatics knowledge, skills, experience, and motivation to drive the initiative forward. In relation to Rule 4a, this approach could be viewed as a progression within an institution once the necessary personnel have been trained. While it is not completely necessary to assess the demand for bioinformatics training in this approach, the integration of a bioinformatics elective into strategically selected undergraduate programs will facilitate the uptake of the course. These selected programs could include students who have developed a strong foundational knowledge in either biology, computer science, mathematics, or statistics. The introduction of bioinformatics electives that expose the students to the complementary application of bioinformatics within each of the previously mentioned disciplines should encourage students to study the electives. The advantages of offering an undergraduate course as part of an existing degree program include exposure of the field to the students to entice potential postgraduate students and the training of a large number of students in basic bioinformatics skills. To ensure that students have a defined academic path to pursue the development of their bioinformatics skills, it is desirable that the introduction of electives be coupled with future planning for the establishment of a formal postgraduate degree program in bioinformatics (Rule 4c). The benefits of introducing bioinformatics courses at the undergraduate level have previously been illustrated and include substantial learning gains in terms of bioinformatics confidence and ability [13] and increased interest and enthusiasm for bioinformatics [14].

### Rule 4c: Set up a formal bioinformatics MSc or PhD degree program

This endeavor will require a coordinated approach from dedicated faculty members who have either developed competencies in bioinformatics or express a strong commitment to do so in the future. The faculty members would require the necessary practical skills to supervise bona fide bioinformatics research projects or initially co-supervise these projects with external experts. The general approach for achieving this will be guided by the rules and guidelines outlined by the institution it is implemented in. As a starting point, if the required expertise is not available within the institution, experienced trainers, mentors, supervisors, and co-supervisors can be sourced from external partner institutions with the aim of developing the necessary skills for local faculty. H3ABioNet has previously been involved in assisting institutions with the development of bioinformatics degree programs within Africa. A general guidelines document for starting a degree program at an institute together with the associated documentation and a recommended bioinformatics postgraduate curriculum has subsequently been developed (https://www.h3abionet.org/training/bioinformaticseducation). These guidelines have successfully been used to implement a number of bioinformatics postgraduate training programs in Africa [1] and include examples of existing curricula within established degree programs, including those from the University of Rhodes, South Africa, and Future University of Sudan. The guidelines cover key steps required to establish a degree program, such as raising awareness of bioinformatics and stakeholder engagement, and also highlight specific issues to consider, such as the program sustainability and alignment with the university’s vision and mission.

### Rule 4d: Create a bioinformatics research group at the faculty level or a national center of excellence

The establishment of a bioinformatics center requires a cadre of independent researchers capable of producing high-quality competitive bioinformatics research. They should have a track record of supervising students and producing original bioinformatics publications and/or software. In addition, they should be able to successfully apply for grants to fund their research. This could start as a single research group led by an experienced bioinformatician. A center of excellence could then be built on this foundation, which, in addition to research, should provide training, education, and bioinformatics support [15,16]. To be successful, a bioinformatics research group or national center of excellence should not only attract and retain researchers and staff members of high caliber, but also develop a long-term plan to leverage local resources and create a sustainable environment, as expanded on in Rules 7 and 9.

### Rule 4e: Build up a bioinformatics service to serve a connected bioinformatics community

While this approach again may be linked to the natural progression and development of bioinformatics skills within an institute, it could also be defined as a specific outcome from the beginning, as mentioned in Rule 4, depending on the local needs. In this instance, an initial investment in hiring one or more senior bioinformatics scientists, with both practical and technical experience in a bioinformatics service environment, is recommended. If this is not possible, the identification and training of suitable candidates could be initiated using the approach mentioned in Rule 4a. Further staff should be recruited to establish a core group with a sufficient array of skills to provide expertise and practical support for a variety of relevant application areas (Rule 8). If this is not possible due to financial limitations, reaching out to other regional support groups with the relevant expertise could be leveraged to initiate collaborations. Integral to the success of a service-oriented unit is the inclusion of staff adequately trained in management skills. This can be developed in house through professional development training of well-selected staff members. From a sustainability point of view, it may also be a fruitful opportunity to partner with an academic institute to allow employees or trainees the opportunity to pursue postgraduate degrees in bioinformatics. It may also be necessary to establish a defined service model incorporating a paid service and/or collaboration option. An obvious requirement for providing a bioinformatics service is the provision and access to significant computing infrastructure to support data analysis [17]. Available options for setting up this infrastructure will be discussed in Rule 6.

## Rule 5: Develop and leverage partnerships with established bioinformatics groups

In all the scenarios described above, it is assumed that some level of local bioinformatics expertise will be available during the process of building up the program. In LMICs, whether in Africa or elsewhere, this may not always be the case. However, programs such as H3ABioNet have already begun to produce well-trained bioinformaticians, limiting the need to source expertise from overseas. In most instances, there will still be a need to provide some level of technical or professional development training to update and acquire new skills. While each of the outcomes or scenarios described above have different focus areas, the success of each scenario hinges on the availability of a few passionate and dedicated local individuals with the relevant skills and a shared vision. An early partnership initiated with the private sector may be beneficial for teaching and training purposes. Specialists in the private sector can be leveraged to provide opportunities for student mentoring through internships, project supervision, seminars, and possible access to funding. This partnership will be beneficial for both parties, as the students will be exposed to the potential application areas of bioinformatics, while the private sector partners can provide some input into the specific bioinformatics skills that are in demand. In a similar way, collaborations between institutions with a joint vision for building bioinformatics capacity can be leveraged to ensure that skills are built as a community.

## Rule 6: Establish and build up computational infrastructure

An integral component of setting up a bioinformatics program for research, training, or service purposes is access to computational resources. The specifications for the infrastructure are dependent on the scale and type of analyses to be run. Small-scale analyses with web-based tools would require access to midrange computers with a reasonable internet connection. This would scale up significantly for the analysis of typically large real-world biological datasets. For this, a medium to high-end server would need to be set up either locally or remotely. From a service perspective, this may be the most suitable solution to ensure adequate computing resources, storage, and backup of data. Investment in physical computing components and the associated infrastructure maintenance is a long-term commitment. In some situations, it may be a viable option to seek collaborations with other academic or strategic partners who may already have access to adequate computational resources. This may include institutional computer science or information and communication technology services departments. A further option, which in some cases may be more practical and cost efficient, is the use of cloud services for compute requirements. There are many cloud service providers available, with the most popular commercial solutions including Amazon Web Services (AWS), Microsoft Azure, and the Google Cloud Platform (GCP). Depending on location and affiliations, there may be some locally available Open Science Cloud services (https://datacarpentry.org/cloud-genomics/04-which-cloud/index.html). In addition to infrastructure, workflows to run common bioinformatics analyses will need to be set up or developed. Several open-source bioinformatics workflows are being developed to facilitate the reproducibility of data analysis methods (https://nf-co.re/pipelines).

## Rule 7: Create communication channels for exposure, feedback, and evaluation

As with any other endeavor, it is necessary to continuously assess progress and adapt to changing needs and requirements. While initially one of the scenarios described in Rules 4a to 4e may apply, over time, it may be necessary to transition to another one. A consistent line of communication and feedback from individuals within the program as well as the larger community will help to ensure that the leadership remains in touch with the developing research areas and service requirements. It may be useful to set up a research seminar series driven by the program and extend invitations to the larger research community [18]. The seminar series can be used to showcase the research areas being explored and help to drive the development of specialized skills, infrastructure, training, and personnel. The series can also be used as an avenue to showcase the work that is being done in the program and encourage networking and collaborations with other scientists [19]. This exposure and local community building can be expanded on social media platforms such as Facebook and Twitter as well as regular newsletter updates as the program develops [20]. Internal needs assessment surveys and training evaluations from attendees should also be conducted regularly, coupled with a means to track skills development as the group increases in size.

## Rule 8: Build up a complementary combination of skilled personnel for your scenario

Each of the described scenarios will require a dedicated individual or group of individuals in order to get started. While it may be feasible to maintain a smaller group at the onset, depending on the long-term goals established through the initial needs assessment process (Rule 1), the development or recruitment of additional group members may be necessary. The desired competencies of these new personnel should be driven by the envisioned trajectory of the newly established bioinformatics program. As previously mentioned in Rule 3, the competencies framework can be used to guide the level of skills required to develop specific bioinformatics personas. In the case of Rule 4a (training existing researchers to analyze data), there may be a need to ensure that those who are trained in specific data analysis techniques are provided with the adequate formal training to ensure that they pass on the knowledge to their colleagues and peers. This might take the form of hosting train-the-trainer events followed by short specialized courses that these individuals can teach. In the academic setting, there may be a need to ensure that the bioinformatics degree programs are diverse enough to expose students to all the relevant areas of bioinformatics. While the academic program at both the undergraduate and postgraduate level may be established by faculty members skilled in a specific area of bioinformatics, over time, it will be useful to either actively develop or recruit faculty members with competencies and skills in other areas. If the initial academic program is set up in one faculty, cross faculty collaborations may lead to the development of additional skills and personas. Development of an undergraduate or postgraduate bioinformatics curriculum within the biology faculty might limit the competency level of the students to only being able to analyze data, while inclusion of members from the computer science or engineering faculty may help to broaden that competency level to the ability to design and develop new bioinformatics tools and resources. The increased diversity of the academic program will attract high-caliber students and also ensure that the course curricula remains up-to-date and relevant with the rapidly developing field. In the context of an established research center, as mentioned in Rule 4d, the growth of the group will be driven by the research output and the ability of the center to attract exceptional academics in the form of independently funded junior and established researchers. The acquisition and availability of funding for postdoctoral researchers in exciting areas of interest will be integral to establishing and building a reputable research center. Similarly, the setting up of a bioinformatics service-orientated environment is heavily reliant on sourcing the correct personnel as has been explained in Rule 4e. As the team grows, there will be a need to ensure that there is a balance in the skills of the personnel available to carry out the various services offered. While some of these skills may not be required at first, there will be a need for personnel to develop skills on data management, ethics, and the concept of making data Findable, Accessible, Interoperable, and Reusable in order to provide useful insights into successful project design. Common and integral to the success of both a research and service-orientated scenario is the need for support staff in the form of project and finance managers and administration assistants. Overall, the success of each scenario is reliant on the local availability of appropriately skilled individuals. It is also important to remain cognizant of diversity and inclusion when building the team.

## Rule 9: Develop a sustainability/progression plan

While it may not be a pressing concern at the start of the program, there will be a need to consider the long-term sustainability of each of the scenarios outlined in Rule 4. As previously mentioned, there may be a natural progression from one scenario to another provided that funding streams are actively obtained to support this. For example, the establishment of undergraduate bioinformatics electives may be implemented within the longer-term context of building up significant capacity to create a postgraduate bioinformatics program. In this case, it would be imperative to ensure that there are sufficient active opportunities to provide additional bioinformatics as well as personal development training to teaching staff to ensure that they will be well equipped to successfully design and supervise MSc and subsequent PhD student projects. This may also entail leveraging support directly from university structures to fully or partially fund tenure positions specializing in bioinformatics together with external funding acquisition. It is very important, as mentioned in Rule 1, to ensure that there is a continual demand for skilled bioinformaticians, especially in the local environment where the program is being set up. While the local demand may fluctuate over time, filling a required skills gap in both industry and academic environments will be the driving force for the continuation of any academic program. In the case of a service-orientated environment, once skilled staff have been employed, internship programs could be set up to encourage local postgraduate students to gain hands-on experience in the data analysis or tool development sector, ensuring that opportunities exist locally to prevent potential new employees from leaving the country for other overseas opportunities. An open communication channel between the academic and the private sector is imperative to prevent “brain-drain” and the loss of valuable locally trained individuals and to ensure the sustainability of any bioinformatics program.

## Rule 10: Embrace local challenges to develop innovative solutions

As is the case with building capacity in any subject area, there are local limitations that may need to be overcome to be successful. As mentioned in Rule 2, obtaining funding specifically for building bioinformatics capacity may be a challenge in both LMICs and high-income countries (HICs). While government bodies and other established institutions and societies may be ready and willing to support bioinformatics capacity development in HICs, in LMICs, this may be much more challenging due to more pertinent areas such as pathogen and agricultural research taking precedence. However, in recent years, projects such as H3Africa and H3ABioNet have successfully demonstrated the value of developing diverse sets of skills across a community rather than conducting research in individual silos. In particular, most African countries are now aware of the value of developing bioinformatics skills across all areas of biological research and are more likely to be supportive of projects aimed at building regional specialized bioinformatics capacity. While training in LMICs has been a challenge due to the lack of expertise and other infrastructural issues, this has given rise to the development and successful presentation of several mixed-model training events that have allowed for the training of large audiences on introductory and intermediate bioinformatics topics [11,12,21]. Through the use of containerized workflows in training events, infrastructure has been set up at several sites in Africa for specific data analysis methods [8,12]. Coupled with the dramatic increase in data produced in LMICs, there has been a major development of Open Science projects aimed at encouraging scientists to both contribute to and utilize Open Data resources [22]. While there is still some hesitancy around data sharing in LMICs, joining such initiatives can provide bioinformatics groups with limited resources access to valuable datasets and additional open-source tools. In addition to capacity development, as mentioned in Rule 4, whatever scenario is selected, there will be a need to either build up or access computational resources. There are several suggestions provided for setting up these resources; however, this may be a very realistic limitation in the sustainability of the program. While a cloud-based system may be a short-term solution, there may be a need to work on a long-term plan for building up local computational resources, especially if you plan to continue to take on more personnel and attract grants involving large-scale studies. As mentioned in Rule 6, seeking collaborations within and outside of your local institute may be the best approach to leverage shared resources to address this issue. While the access to computational resources may be the initial reason for collaboration, the interaction can be used to explore joint ventures to secure funding for further development of the shared computational resources even if the research areas are completely different. In terms of sourcing skilled personnel, if individuals are not being trained at a local institution, it is helpful to be aware of the various training programs in the region. In Africa, there are several Fogarty-funded postgraduate programs currently running, which produce well-equipped MSc and PhD graduates in addition to several other independent bioinformatics postgraduate programs [1,4]. Junior academic and service staff can be sourced from these programs to assist with developing any of the scenarios described in Rule 4, and there may not be a need to look overseas for skilled personnel.

## Conclusions

While setting up any new program, whether academic or service orientated, is a challenging task, the rules outlined in this article provide a practical thought process to follow, together with some established resources that will aid in the development of a bioinformatics program. In an era driven by data science, the need for bioinformatics research and service activities within academic institutes, especially in LMICs, is essential to ensure equal opportunities for competitive research funding. The need to realistically match the level of bioinformatics support requirements of an institution to the type of program it would like to develop provides a logical and critical way to start a tailored bioinformatics program in any setting. Leveraging the experience and resources of well-established groups such as H3ABioNet and others provides a springboard to kick-start the establishment of such a program. We hope that this framework will provide the necessary encouragement and resources for anyone who is thinking about starting up a bioinformatics program at their institute to dive in and get started.
